# Noninvasive prediction of glypican-3 expression and recurrence-free survival in hepatocellular carcinoma using contrast-enhanced ultrasound

**DOI:** 10.1186/s40644-026-01033-9

**Published:** 2026-04-11

**Authors:** Lianhua Zhu, Wenbo Tang, Bo Jiang, Peng Han, Miao Li, Jinrui Zhu, Xiang Fei

**Affiliations:** 1 Department of Ultrasound, The First Medical Center, Chinese PLA General Hospital, Beijing, 100853 China; 2 Department of Ambulatory Medicine, The First Medical Center, Chinese PLA General Hospital, Beijing, 100853 China

**Keywords:** Hepatocellular carcinoma, Glypican-3, Contrast enhanced ultrasound, Nomogram, Recurrence-free survival

## Abstract

**Background:**

Glypican-3 (GPC-3) is a promising biomarker and therapeutic target in hepatocellular carcinoma (HCC), yet its noninvasive preoperative assessment remains challenging. This study developed and validated a nomogram model integrating contrast-enhanced ultrasound (CEUS) features with clinical variables to predict GPC-3 expression and assess its prognostic significance for recurrence-free survival (RFS).

**Methods:**

A retrospective analysis of 316 HCC patients who underwent preoperative CEUS was performed. Predictive CEUS features and clinical variables were identified using least absolute shrinkage and selection operator regression and multivariable logistic regression. The nomogram was constructed and validated in training and validation cohorts, with performance assessed by receiver operating characteristic curve, calibration curves, and decision curve analysis. Kaplan-Meier and Cox regression analyses were used to assess the association between GPC-3 expression, nomogram scores, and RFS.

**Results:**

The median age of the patients was 59 years (IQR, 52–67 years), with a predominance of male patients (267/316, 84.49%). Four predictors of GPC-3 expression were identified: alpha-fetoprotein, feeding artery, hyper-enhanced rim in the arterial phase, and necrosis. The nomogram achieved area under the curve values of 0.90 (95% CI: 0.86, 0.94) in the training cohort and 0.88 (95% CI: 0.80, 0.94) in the validation cohort. The positive predictive value and negative predictive value were 97.14% (95% CI: 94.38, 99.90) and 54.32% (95% CI: 43.47, 65.17) in the training cohort, and 96.77% (95% CI: 91.80, 99.99) and 45.46% (95% CI: 28.12, 62.08) in the validation cohort, respectively. Calibration plots and decision curve analysis confirmed its clinical utility. GPC-3 expression was identified as an independent prognostic factor for RFS (hazard ratio: 3.56; 95% CI: 2.18, 5.83), and patients with high nomogram scores had significantly shorter RFS (*P* < 0.001).

**Conclusions:**

This CEUS-based nomogram provides an accurate, noninvasive method for predicting GPC-3 expression and RFS in HCC patients, serving as a valuable tool for personalized treatment strategies and optimizing GPC-3-targeted therapies.

**Supplementary Information:**

The online version contains supplementary material available at 10.1186/s40644-026-01033-9.

## Background

Hepatocellular carcinoma (HCC) accounts for 85–90% of primary liver cancer cases, representing one of the most prevalent and challenging malignancies worldwide [1, 2]. Despite advances in therapeutic strategies, the prognosis for HCC remains poor, with surgical resection being the cornerstone of curative treatment. However, this option is limited to patients diagnosed at an early stage [3]. Even after curative resection, recurrence rates are alarmingly high, reaching up to 70% within five years [4]. These challenges underscore the urgent need for reliable biomarkers to improve early diagnosis, guide treatment decisions, and refine prognosis assessments.

Glypican-3 (GPC-3) has emerged as a promising biomarker and therapeutic target in HCC, being selectively expressed in 75–90% of cases and minimally in healthy liver tissue [5]. GPC-3 is integral to tumor initiation, proliferation, and metastasis in HCC. Elevated GPC-3 expression has been strongly associated with poor clinical outcomes, including reduced overall survival and disease-free survival [6, 7]. Furthermore, recent clinical trials have demonstrated that GPC3-targeted therapy can effectively inhibit HCC growth [5, 8]. Consequently, detecting GPC-3 expression is essential for optimizing treatment plans and evaluating prognosis. However, current assessment methods, such as immunohistochemistry, require invasive tissue samples obtained via surgical resection or biopsy [9]. Developing a noninvasive, preoperative method to evaluate GPC-3 expression would significantly enhance clinical decision-making and prognosis prediction, offering profound implications for patient management in HCC.

Contrast-enhanced ultrasound (CEUS) is a highly versatile imaging modality that combines high temporal and spatial resolution with the ability to visualize tumor perfusion and microcirculation in real-time [10, 11]. CEUS has demonstrated significant utility in the early diagnosis of HCC and the assessment of therapeutic responses [12, 13]. Additionally, emerging evidence suggests that CEUS features may reflect the biological behavior of HCC, including microvascular invasion and Ki-67 expression [14–16]. However, the potential of CEUS to predict GPC-3 expression in HCC remains largely unexplored. Establishing this relationship could pave the way for a noninvasive imaging-based approach to evaluate this critical biomarker preoperatively and assess prognosis.

This study aims to address this knowledge gap by developing a nomogram model that integrates clinical features and CEUS characteristics to evaluate GPC-3 expression in HCC before surgery. Additionally, we explore the utility of the nomogram model in predicting patient prognosis, specifically recurrence-free survival (RFS). By establishing a robust imaging-based method for predicting GPC-3 expression and prognosis, this study seeks to advance precision oncology in HCC management and improve patient outcomes.

## Methods

### Study population

**Fig. 1:**
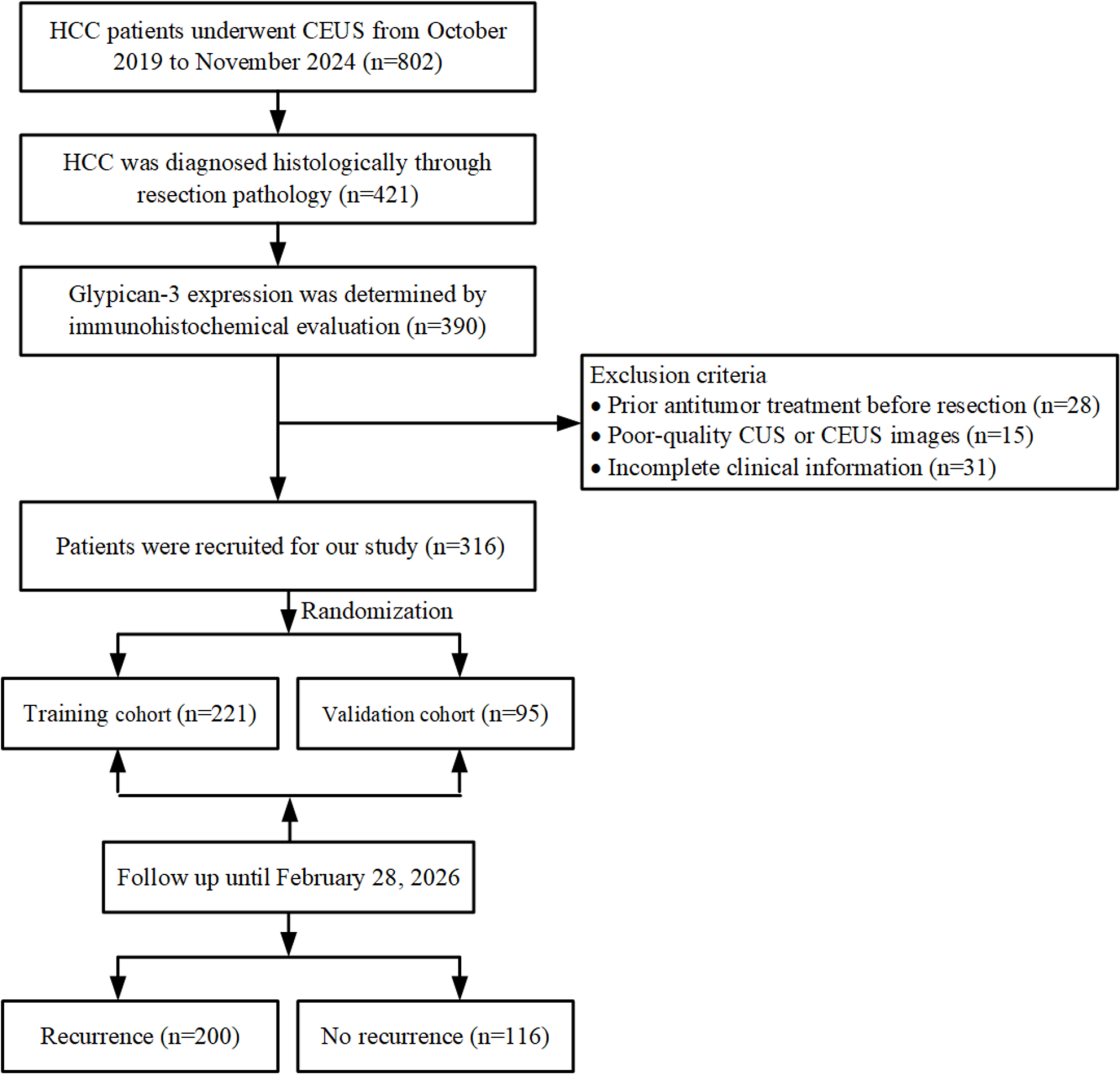
Flowchart of patient selection. HCC = hepatocellular carcinoma, CEUS = contrast-enhanced ultrasound, CUS = conventional ultrasound

This retrospective study was conducted with the approval of the Ethics Committees of Chinese PLA General Hospital (Clinical trial number: not applicable), in accordance with the Declaration of Helsinki, with informed consent waived due to retrospective nature of the study. From October 2019 to November 2024, 802 patients diagnosed with HCC who underwent CEUS examinations were screened for eligibility. Inclusion criteria were: (1) preoperative CEUS examination performed within two weeks before surgery; (2) histological confirmation of HCC based on postoperative resection pathology; and (3) immunohistochemical evaluation of GPC-3 expression. Exclusion criteria included: (1) prior antitumor treatments before surgical resection; (2) poor-quality conventional ultrasound (CUS) or CEUS images; and (3) incomplete clinical data. Ultimately, 316 patients were enrolled (Fig. 1).

### Ultrasound equipment and scanning protocol

Ultrasound examinations were performed using Sequoia (Siemens) and Resona 7/R9 (Mindray) systems with 5C1 and SC5-1U convex transducers, respectively, set at 2.0 to 5.0 MHz. CUS and CEUS were performed in accordance with the guidelines of the European Federation of Societies for Ultrasound in Medicine and Biology [17]. The purpose of CUS was to examine the tumor lesions and the surrounding liver parenchyma. CEUS was subsequently performed using a 2.4 mL bolus injection of SonoVue (Bracco), with real-time dynamic enhancement observed for at least 30 s during the arterial phase, and intermittent scanning until 5 min post-injection. The mechanical index was maintained between 0.06 and 0.09.

### Clinical information collection

Demographic and clinical data, including age, sex, HCC risk factors (hepatitis B and C infections, steatohepatitis, alcohol abuse, cirrhosis), status (primary and recurrent), Child−Pugh classification, Barcelona Clinic Liver Cancer stage, and preoperative alpha-fetoprotein (AFP) levels, were extracted from medical records.

### Ultrasound image analysis

All ultrasound images were independently reviewed by two experienced physicians (L.Z. and X.F.), each with over 10 years of expertise in abdominal CEUS, who were blinded to pathological and clinical data. Interobserver reliability was assessed using intraclass correlation coefficients or kappa statistics. Discrepancies in the interpretation of ultrasound image features were resolved through discussion, and a consensus was reached. For patients with multiple tumors, the largest lesion was selected for assessment. The analyzed CUS features included tumor location, number, echogenicity (relative to adjacent liver parenchyma, hypoechoic, isoechoic, or hyperechoic), boundary (clear or unclear), morphology (regular or irregular), maximum diameter, and blood flow presence assessed using color Doppler imaging.

The analyzed features of CEUS imaging included initial enhancement time (relative to adjacent liver parenchyma, earlier or not earlier), hyper-enhanced rim (2–3 mm hyperenhancement band-shaped areas observed around the tumor lesion during the arterial phase, present or absent), peak intensity in the arterial phase (relative to adjacent liver parenchyma, hyperenhancement or non-hyperenhancement), feeding artery (present or absent), enhancement mode (homogeneous or heterogeneous), necrosis (non-enhancing area within the tumor lesion, present or absent), portal early and late enhancement intensities (relative to adjacent liver parenchyma, hypo-enhancement or non-hypo-enhancement), washout in the delayed phase (relative to adjacent liver parenchyma, marked or mild) [18, 19]. The CEUS enhancement process was segmented into four distinct phases: arterial phase (10–30 s), portal early phase (30–60 s), portal late phase (60–120 s), and delayed phase (120–300 s) [17].

### Pathological analysis

Histological examination of resected specimens confirmed HCC diagnosis. Immunohistochemical staining evaluated GPC-3 expression, with positive expression defined as ≥ 5% staining positivity with moderate-to-strong staining intensity [20, 21].

### Follow up

Postoperative follow-up included imaging assessments with contrast-enhanced CT/MRI or abdominal ultrasound, alongside laboratory testing (serum AFP levels). Patients were monitored at one month post-resection and every 4–6 months thereafter until recurrence, death, or the study end (February 28, 2026). RFS was defined as the time from surgical resection to the earliest event, including tumor recurrence (confirmed by imaging), death from any cause, or the last follow-up.

### Statistical analysis

Statistical analyses were performed using R software (version 4.4.3). Continuous variables were expressed as medians and interquartile ranges (IQRs), while categorical variables as frequencies and percentages. Patients were divided into training (70%) and validation cohorts (30%) using stratified randomization based on GPC-3 expression. Predictors of GPC-3 expression were identified using multivariable least absolute shrinkage and selection operator regression and further analyzed with multivariable logistic regression to construct a predictive model, presented as a nomogram. The optimal lambda value for least absolute shrinkage and selection operator regression was determined using 10-fold cross-validation, where lambda.min, the value that minimizes the cross-validated mean-squared error, was selected as the final parameter to ensure optimal model performance. Model fit was assessed using the Akaike information criterion and the Hosmer-Lemeshow goodness-of-fit test [22]. Nomogram model discrimination was evaluated using the area under the receiver operating characteristic curve (AUC), and calibration with calibration plots. The cutoff score for the nomogram was determined using Youden’s index. The DeLong test was used to compare the AUC of the nomogram model with those of independent predictors. Clinical utility was determined through decision curve analysis (DCA). Independent prognostic factors for RFS were identified using multivariable Cox proportional hazards regression analysis, reported as hazard ratio and 95% CI for the entire patient cohort. The prognostic value of the nomogram model and GPC-3 expression for RFS was evaluated using Kaplan-Meier survival analysis, with group differences tested using the log-rank test. Statistical significance was defined as a *P*-value < 0.05.

## Results

### Patient baseline characteristics

This study included 316 patients who underwent surgical resection for HCC. The median age was 59 years (IQR, 52–67 years), with a predominance of male patients (267/316, 84.49%). GPC-3 expression was identified in 79.43% (251/316) of the HCCs. Hepatitis B infection was the most common risk factor (252/316, 79.75%), followed by hepatitis C infection (28/316, 8.86%), nonalcoholic steatohepatitis (15/316, 4.75%), and alcohol abuse (14/316, 4.43%). Additionally, 211 patients (66.77%) had cirrhosis. Primary HCC accounted for 94.30% (298/316) of cases. Most patients had well-preserved liver function, with 96.84% (306/316) classified as Child-Pugh A and 3.16% (10/316) as Child-Pugh B. According to the Barcelona Clinic Liver Cancer stage, 15.51% (49/316) of patients were in stage 0, 22.78% (72/316) in stage A, and 61.71% (195/316) in stage B. Preoperative serum AFP levels had a median value of 11.92 µg/L (IQR, 3.22–162.68 µg/L), with 44.62% (141/316) of patients presenting AFP levels above 20 µg/L. Baseline characteristics were compared between the training and validation cohorts, showing no statistically significant differences (*P* > 0.05), indicating balanced cohort distribution (Table 1). Table S1 presents the clinical characteristics of patients stratified by GPC-3 expression.

**Table 1:** Clinicopathologic characteristics of patients in the training and validation data sets

Characteristic	Training cohort (*n* = 221)	Validation cohort (*n* = 95)	*P* Value
Age (years)	58 (51–67)	61 (55–67)	0.07
Sex			0.67
Male	188 (85.07%)	79 (83.16%)	
Female	33 (14.93%)	16 (16.84%)	
Hepatitis B			0.82
Present	177 (80.09%)	75 (78.95%)	
Absent	44 (19.91%)	20 (21.05%)	
Hepatitis C			0.50
Present	18 (8.14%)	10 (11.53%)	
Absent	203 (91.86%)	85 (89.47%)	
Steatohepatitis			0.40
Present	9 (4.07%)	6 (6.32%)	
Absent	212 (95.93%)	89 (93.68%)	
Alcoholism			0.57
Present	11 (4.98%)	3 (3.16%)	
Absent	210 (95.02%)	92 (96.84%)	
Cirrhosis			0.88
Present	147 (66.52%)	64 (67.37%)	
Absent	74 (33.48%)	31 (32.63%)	
Status			0.83
Primary	208 (94.12%)	90 (94.74%)	
Recurrent	13 (5.88%)	5 (5.26%)	
Child-Pugh			0.73
A	213 (96.38%)	93 (97.89%)	
B	8 (3.62%)	2 (2.11%)	
Barcelona Clinic Liver Cancer			0.17
0	38 (17.19%)	11 (11.58%)	
A	54 (24.43%)	18 (18.95%)	
B	129 (58.37%)	66 (69.47%)	
Alpha-fetoprotein (µg/L)	11.46 (3.11–192.50)	14.29 (3.68–113.70)	0.98
Glypican-3			0.44
Positive	173 (78.28%)	78 (82.11%)	
Negative	48 (21.72%)	17 (17.89%)	

Note: Quantitative data are median (interquartile range)

### Predictor selection in the training cohort

**Fig. 2:**
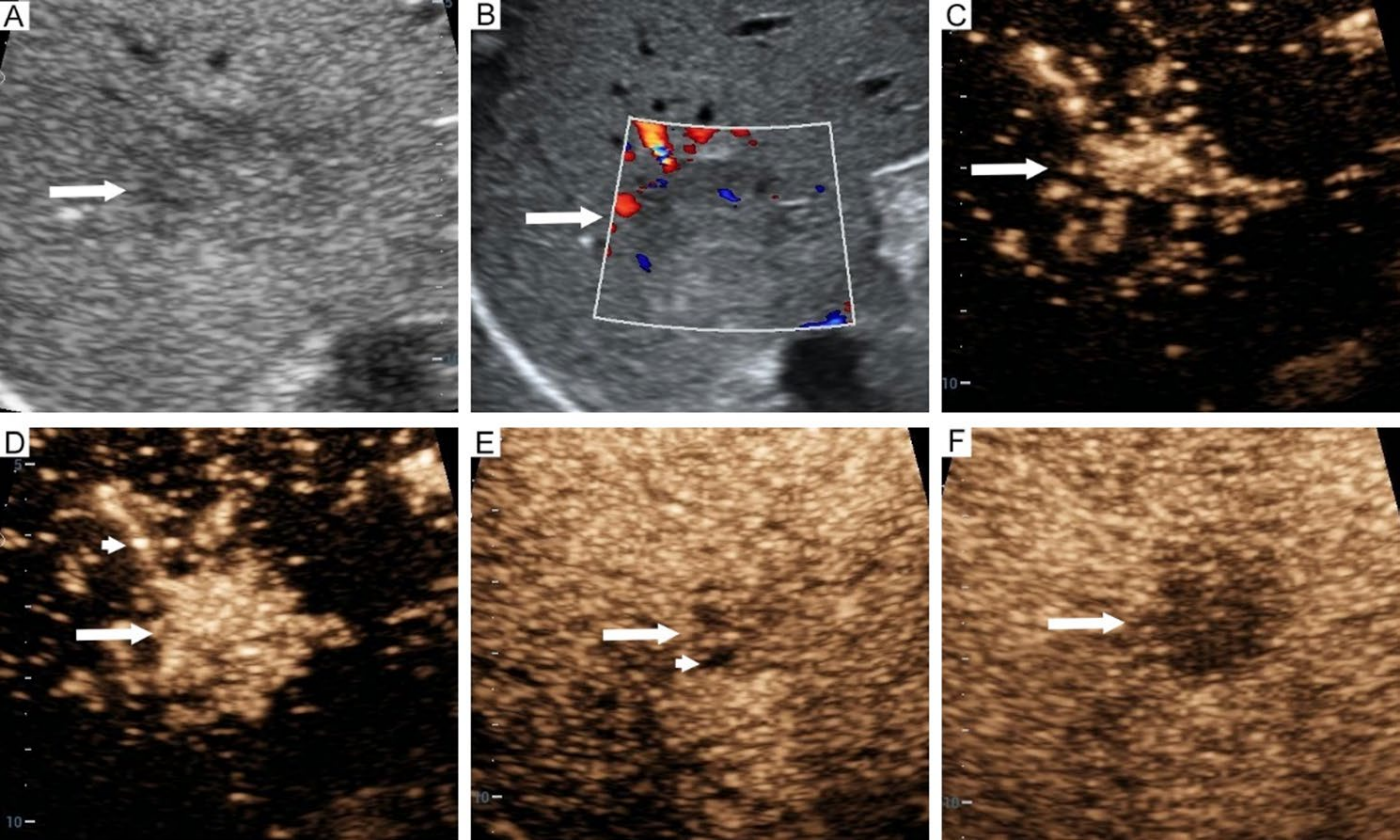
Imaging features of glypican-3-positive hepatocellular carcinoma in a 42-year-old man. Isoechoic lesion in segment 7 (**a)**. Blood flow in the lesion (**b)**. Contrast-enhanced ultrasound reveals early enhancement at 11s (**c**), feeding artery (arrowhead) at 13s (**d**), and necrosis (arrowhead) at 25s, all within the arterial phase (**e**), followed by hypo-enhancement at 94s in the portal late phase (**f**). Arrow indicates the hepatocellular carcinoma lesion

**Fig. 3:**
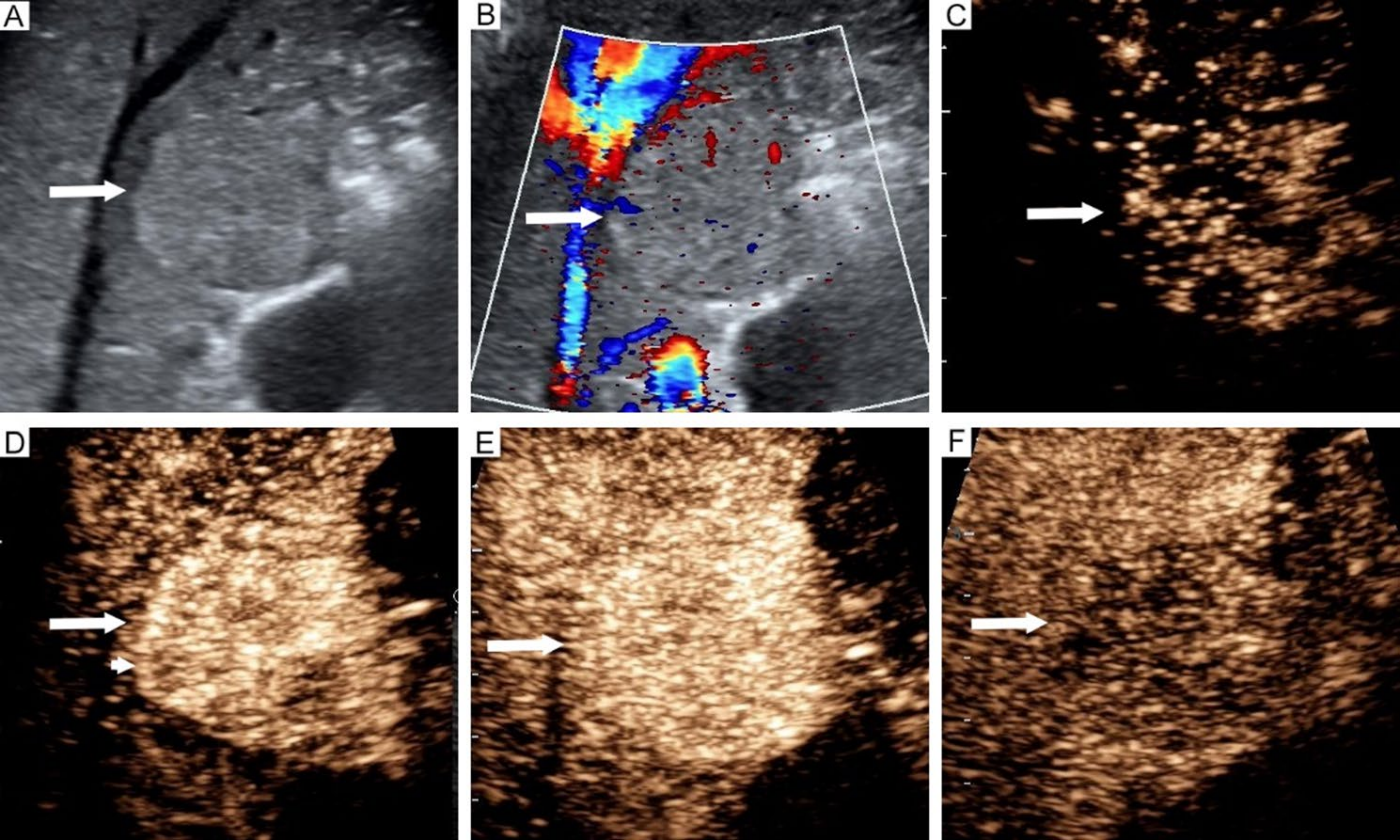
Imaging features of glypican-3-negative hepatocellular carcinoma in a 63-year-old man. Isoechoic lesion in segment 5 (**a)**. Blood flow in the lesion (**b)**. Contrast-enhanced ultrasound reveals early enhancement at 12s (**c**), hyper-enhanced rim (arrowhead) at 15s (**d**), and no necrosis at 21s, all within the arterial phase (**e**), followed by hypo-enhancement at 108s in the portal late phase (**f**). Arrow indicates the hepatocellular carcinoma lesion

**Fig. 4:**
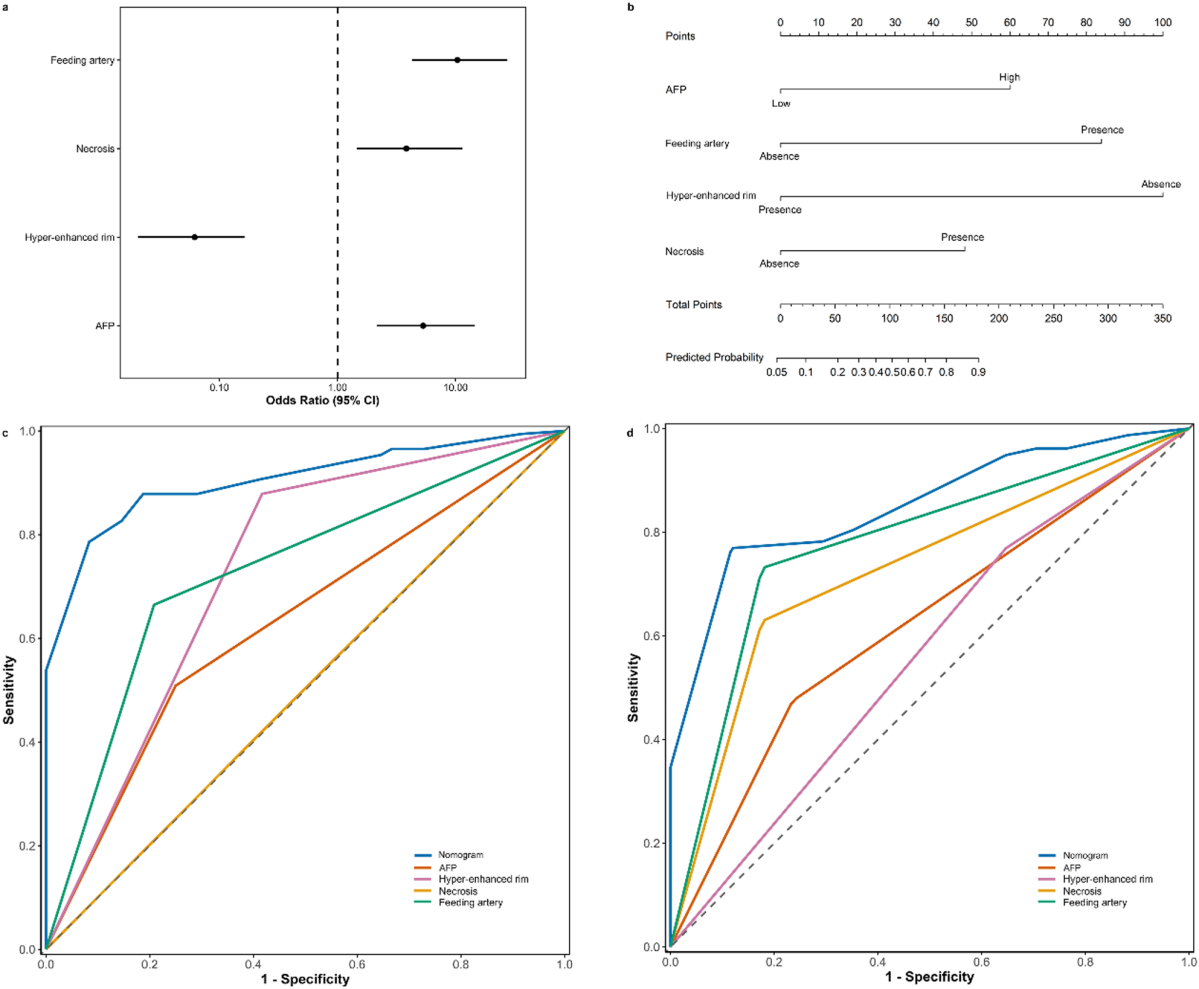
Prediction of glypican-3 expression. Forest plot showing independent predictors of glypican-3 expression (**a**). Nomogram model for predicting glypican-3 expression (**b**). Receiver operating characteristic curves in the training cohort (**c**) and validation cohort (**d**). AFP = alpha-fetoprotein

Least absolute shrinkage and selection operator regression analyzed 27 preoperative variables to identify predictors of GPC-3 expression, retaining 7 significant predictors: hepatitis C (coefficient: 0.05), AFP (coefficient: 0.14), tumor boundary (coefficient: -0.01), feeding artery (coefficient: 0.22), hyper-enhanced rim in the arterial phase (coefficient: -0.34), necrosis (coefficient: 0.06), and Barcelona Clinic Liver Cancer stage (coefficient: 0.004). Multivariable logistic regression further identified 4 independent predictors: AFP, feeding artery, hyper-enhanced rim in the arterial phase, and necrosis (Table 2) (Figs. 2 and 3). Variance inflation factor testing confirmed no multicollinearity among the predictors (variance inflation factor < 2), ensuring strong stability and fit. The contribution of the four independent predictors to GPC-3 expression is illustrated in Fig. 4a.

**Table 2:** Logistic regression analysis of characteristics associated with glypican-3 expression

Variables	Variance inflation factor	Odds ratio (95%CI)	*p* Value
Alpha-fetoprotein	1.14	5.32 (2.16–14.46)	< 0.001
Feeding artery	1.12	10.35 (4.27–27.50)	< 0.001
Hyper-enhanced rim	1.43	0.06 (0.02–0.16)	< 0.001
Necrosis	1.46	3.84 (1.45–11.41)	0.009
Constant		0.94 (0.44–1.99)	0.87

### Nomogram model development and validation

This model was visualized as a nomogram for quantitative risk prediction, with a cutoff score of 172 indicating a greater likelihood of GPC-3 expression (Fig. 4b). The nomogram model demonstrated optimal performance with the lowest Akaike information criterion (154.11) in the training set. The discrimination of the nomogram model and each independent predictor was assessed using the AUC (Table 3) (Fig. 4c and d). In the training cohort, the nomogram model achieved an AUC of 0.90 (95% CI: 0.86, 0.94), with a specificity of 91.67% (95% CI: 79.17, 98.13) and a sensitivity of 78.61% (95% CI: 64.16, 87.86). Similarly, in the validation cohort, the AUC was 0.88 (95% CI: 0.80, 0.94), with a specificity of 88.24% (95% CI: 70.59, 99.99) and a sensitivity of 76.92% (95% CI: 67.08, 86.08). The AUC in training and validation cohort was larger than that of each independent predictor (*P* < 0.05). These results demonstrated the excellent discriminatory and predictive value of nomogram model. Necrosis demonstrated an AUC of 0.50 in the training cohort, which increased to 0.73 in the validation cohort. In contrast, the hyper-enhanced rim showed a decrease in AUC from 0.73 in the training cohort to 0.56 in the validation cohort. The positive predictive value and negative predictive value were 97.14% (95% CI: 94.38, 99.90) and 54.32% (95% CI: 43.47, 65.17) in the training cohort, and 96.77% (95% CI: 91.80, 99.99) and 45.46% (95% CI: 28.12, 62.08) in the validation cohort, respectively.

**Table 3:** The area under the receiver operating characteristic curve for predicting glypican-3 expression

Variables	Training cohort (*n* = 221)	Validation cohort (*n* = 95)
Alpha-fetoprotein	0.63 (0.56–0.70)	0.62 (0.50–0.73)
Feeding artery	0.73 (0.66–0.79)	0.78 (0.68–0.87)
Hyper-enhanced rim	0.73 (0.66–0.81)	0.56 (0.44–0.69)
Necrosis	0.50 (0.42–0.58)	0.73 (0.62–0.82)
Nomogram	0.90 (0.86–0.94)	0.88 (0.80–0.94)

### Nomogram model calibration

**Fig. 5:**
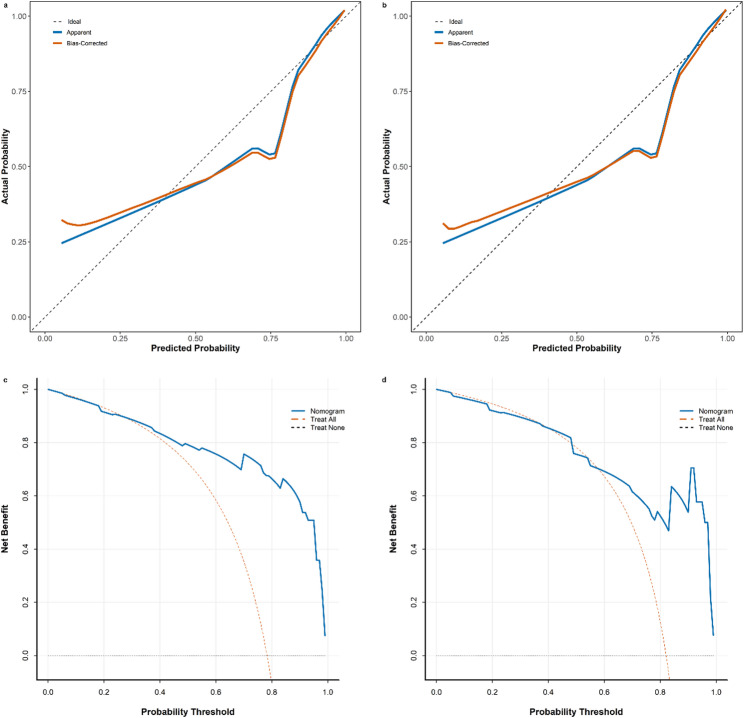
Evaluation of nomogram models. Calibration curve for the training cohort (**a**) and validation cohort (**b**). Decision curve for the training cohort (**c**) and validation cohort (**d**)

Calibration plots and the Hosmer-Lemeshow goodness-of-fit test were used to evaluate the performance of the nomogram model. The results showed good calibration in both cohorts, with the Hosmer-Lemeshow test indicating no significant deviation between predicted and observed probabilities (*P* = 0.18 and 0.16, respectively). Calibration plots indicated strong agreement between predicted and observed probabilities of GPC-3 expression (Fig. 5a and b).

### Clinical evaluation of the nomogram model

The clinical utility of the nomogram model was assessed using DCA, as illustrated in Fig. 5c and d. DCA demonstrated that the nomogram model provided significantly higher net benefit compared to treat-all and treat-none scenarios across a wide range of threshold probabilities in both cohorts. This indicated that the nomogram model effectively balances the risks of overtreatment and undertreatment, offering superior clinical utility.

### Predictive value of the nomogram for RFS

**Fig. 6:**
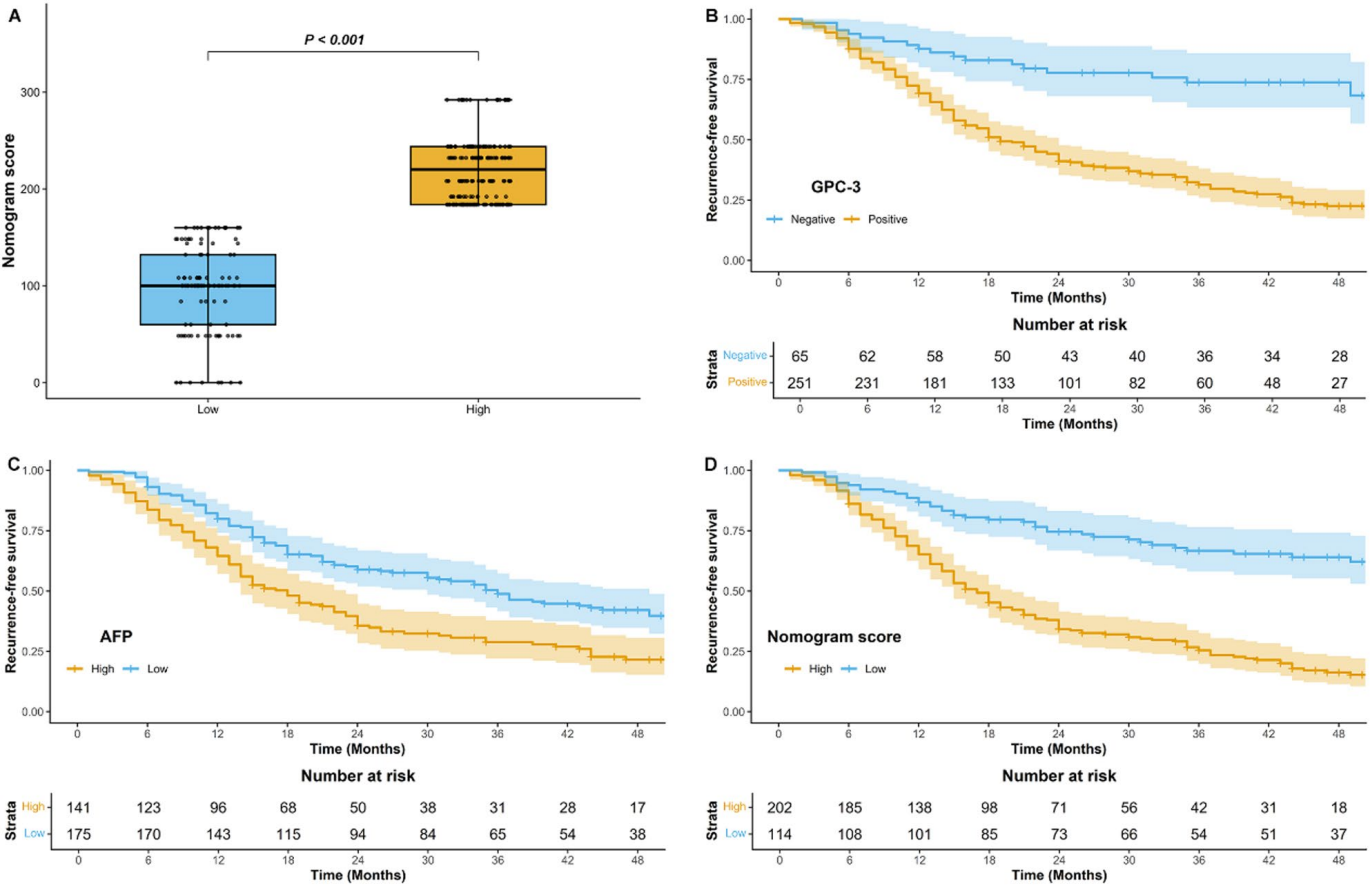
Prognostic value of the nomogram. Box plot comparing the nomogram scores (**a**). Kaplan-Meier curves of recurrence-free survival according to glypican-3 expression (**b**), AFP level (**c**), and nomogram score (**d**). GPC-3 = glypican-3, AFP = alpha-fetoprotein

The median follow-up duration for the survival analysis cohort was 21 months (IQR, 12–43 months). Of the 316 patients, 200 (63.29%) experienced recurrence, while 116 (36.71%) remained recurrence-free. Patients were stratified into high and low nomogram score groups based on the cutoff score, with significantly higher risk in the high-score group (*P* < 0.001) (Fig. 6a). Multivariable Cox regression analysis identified GPC-3 expression (hazard ratio: 3.56; 95% CI: 2.18, 5.83; *P* < 0.001) and AFP level > 20 µg/L (hazard ratio: 1.55; 95% CI: 1.17, 2.06; *P* = 0.002) as independent prognostic factors for RFS. Kaplan-Meier survival curves revealed significant differences in RFS based on GPC-3 expression and AFP level (*P* < 0.001) (Fig. 6b and c). Additionally, patients with high nomogram scores (> 172) demonstrated significantly shorter RFS compared to those with low scores (≤ 172) (*P* < 0.001) (Fig. 6d).

### Interobserver agreement

Interobserver agreement for CUS and CEUS image features showed moderate to excellent agreement across different characteristics (coefficient = 0.66–0.87) (Table 4). The agreement in tumor maximum diameter was the highest (coefficient = 0.87, 95% CI: 0.81, 0.91), while initial enhancement time and peak intensity in the arterial phase was the lowest (κ = 0.66, 95% CI: 0.04, 1.00).

**Table 4:** Interobserver agreement for conventional ultrasound and contrast-enhanced ultrasound image features

Characteristics	Interobserver agreement	95% CI
Tumor maximum diameter	0.87	0.81–0.91
Echogenicity	0.82	0.76–0.89
Boundary	0.79	0.66–0.91
Morphology	0.78	0.66–0.91
Blood flow	0.73	0.54–0.92
Initial enhancement time	0.66	0.04-1.00
Peak intensity in the arterial phase	0.66	0.04-1.00
Feeding artery	0.82	0.70–0.94
Hyper-enhanced rim in the arterial phase	0.79	0.65–0.93
Necrosis	0.77	0.64–0.90
Enhancement mode	0.74	0.59–0.88
Portal early enhancement intensity	0.75	0.59–0.90
Portal late enhancement intensity	0.76	0.62–0.90
Washout in delayed phase	0.80	0.65–0.94

## Discussion

This study successfully developed and validated a nomogram model integrating CEUS imaging features and clinical parameters for noninvasive preoperative assessment of GPC-3 expression in HCC. The nomogram model, constructed based on AFP levels, feeding artery presence, hyper-enhanced rim in the arterial phase, and necrosis, demonstrated excellent discrimination performance in both training and validation cohorts. Furthermore, GPC-3 expression was confirmed as an independent prognostic factor for RFS, with higher nomogram scores associated with shorter RFS. These findings highlight the clinical significance of this nomogram model in HCC management, bridging imaging and molecular diagnostics to advance precision oncology.

GPC-3 has been established as a biomarker and therapeutic target in HCC due to its selective expression and critical role in tumor initiation, proliferation, angiogenesis, and metastasis [5, 23]. Previous studies have linked GPC-3 expression to aggressive tumor phenotypes and poor clinical outcomes, and our results corroborate these findings [7]. Kaplan-Meier survival analysis revealed that patients with positive GPC-3 expression had significantly shorter RFS compared to those with negative expression. Multivariable Cox regression analysis further confirmed GPC-3 as an independent prognostic factor for RFS. GPC-3 promotes tumor angiogenesis through influencing the expression of vascular growth factors and the activity of signaling pathways, suggesting microcirculation perfusion is related to GPC-3 expression [24, 25].

CEUS is a dynamic, real-time imaging modality that provides detailed assessments of tumor microcirculation perfusion. This study identified feeding artery presence, hyper-enhanced rim in the arterial phase, and necrosis as independent predictors of positive GPC-3 expression in HCC, providing new insights into the relationship between imaging features and molecular biomarkers. Feeding artery, a branch of the hepatic artery, supplies nutrition and oxygen to rapidly growing tumors [26]. This feature reflects the tumor’s high metabolic activity and vascular development, driven by GPC-3-induced angiogenesis. The presence of feeding artery was observed more frequently in HCC with positive GPC-3 expression, consistent with previous studies [19]. CEUS enables accurate detection of feeding arteries in HCC, emphasizing its value in preoperative evaluation. Hyper-enhanced rim in the arterial phase is caused by the compression of normal liver tissue surrounding HCC due to expansive tumor growth. In this study, the absence of hyper-enhanced rim was more frequently observed in GPC-3-positive tumors, potentially indicating barrier breakdown and the spread of tumor cells, which are risk factors for invasion and metastasis [27]. This finding aligns with previous studies using MRI [28]. As GPC-3 promotes tumor growth and metastasis, hyper-enhanced rim features become less prominent, making their absence a valuable predictor. Tumor necrosis reflects hypoxic conditions within the tumor microenvironment, driving tumor heterogeneity and promoting aggressive phenotypes. GPC-3-positive tumors may exhibit increased susceptibility to hypoxia-induced necrosis due to their rapid growth and metabolic demands. This study found that necrosis was more frequently detected in tumors with positive GPC-3 expression, consistent with prior findings [25]. CEUS, through its ability to visualize tumor microcirculation, provides critical insights into necrosis patterns and their association with molecular features like GPC-3 expression. These findings underscore the utility of CEUS imaging in identifying key predictors of GPC-3 expression, offering a noninvasive and clinically applicable option for preoperative assessment [29].

AFP is a well-established biomarker for HCC, often associated with tumor progression, metastasis, and poor clinical outcomes. In this study, elevated AFP levels (> 20 µg/L) were identified as another key predictor of positive GPC-3 expression. Previous studies have demonstrated the synergistic roles of AFP and GPC-3 in regulating tumor growth and progression [6, 21, 30]. Additionally, GPC-3 and AFP may share common transcription factors during tumorigenesis, further linking AFP levels to GPC-3 expression [31]. Beyond its predictive value, elevated AFP levels and positive GPC-3 expression were significantly associated with increased HCC recurrence risk, emphasizing their importance as biomarkers for tumor aggressiveness and prognosis.

By integrating CEUS imaging features with AFP levels, our proposed nomogram model demonstrates significant clinical utility in preoperative identification of HCC tumors expressing GPC-3. The predictive performance of our model is comparable to that of magnetic resonance imaging [21, 28], suggesting that CEUS is a valuable complementary imaging modality for predicting GPC-3 expression. This is particularly beneficial for patients with renal impairment who may be unable to undergo magnetic resonance imaging [11, 12]. This model can inform optimized surgical strategies and enable closer postoperative monitoring. Notably, GPC-3, a promising target for immunotherapy, could allow patients with positive expression to benefit from tailored adjuvant therapies, potentially improving their outcomes [32].

Despite its strengths, this study has several limitations. First, the retrospective, single-center design may introduce selection bias and restrict the generalizability of the nomogram model. The findings may not fully represent diverse populations or clinical settings, particularly where CEUS is not routinely used. Prospective validation in multicenter cohorts is essential to confirm the robustness of the nomogram model. Second, the median follow-up time of 21 months may be insufficient to capture long-term survival outcomes. Extended follow-up periods, such as 5 years, are needed to better evaluate the prognostic value of the nomogram model. Third, discrepancies in the performance of individual predictors, such as necrosis and hyper-enhanced rim, were observed. These variations are likely due to differences in sample distribution and the smaller size of the validation cohort, which is an inherent limitation of single-feature analysis. However, the nomogram model, which integrates multiple predictors, exhibited stable and robust performance across cohorts, mitigating the impact of individual predictors fluctuations. Future research should focus on larger and more diverse datasets, as well as advanced feature selection techniques, to enhance the stability and predictive power of individual predictors. Lastly, this study only included patients who underwent surgical resection, enabling the acquisition of complete pathological specimens for accurate assessment of GPC-3 expression. However, this design may overrepresent early-stage tumors (96.8% were Child-Pugh A) and limit the applicability of findings to patients with advanced-stage HCC or those undergoing non-surgical treatments, such as locoregional or systemic therapies. As GPC-3 expression may vary across disease stages, future studies should incorporate more diverse cohorts with varying clinical presentations and treatment strategies to validate the prognostic utility of GPC-3 expression in broader clinical contexts.

## Conclusions

In summary, this study presents an innovative nomogram model for preoperative evaluation of GPC-3 expression in HCC patients. By integrating CEUS imaging features and AFP levels, the nomogram effectively predicts GPC-3 expression and recurrence risk. This model bridges imaging and molecular diagnostics, advancing precision oncology and providing a noninvasive tool for individualized treatment planning. Furthermore, its ability to predict GPC-3 expression could aid in identifying patients suitable for investigational immunotherapies, potentially guiding future treatment strategies as these therapies continue to evolve. This study paves the way for more tailored and effective management strategies in clinical practice, thereby improving outcomes for HCC patients.

## Supplementary Information

Below is the link to the electronic supplementary material.

Supplementary Material 1

## Data Availability

The data that support the findings of this study are available from the corresponding author upon reasonable request.

## Abbreviations

AFP: Alpha-fetoprotein
AUC: Area under the receiver operating characteristic curve
CEUS: Contrast-enhanced ultrasound
CUS: Conventional ultrasound
DCA: Decision curve analysis
GPC-3: Glypican-3
HCC: Hepatocellular carcinoma
RFS: Recurrence-free survival
