# Supplementary material for: Noninvasive prediction of glypican-3 expression and recurrence-free survival in hepatocellular carcinoma using contrast-enhanced ultrasound
Source: Cancer Imaging. 2026 Apr 11;26:67. doi: 10.1186/s40644-026-01033-9 (PMC13182096; doi:10.1186/s40644-026-01033-9)
Supplement: Supplementary file 1 — Supplementary Material 1 [file 40644_2026_1033_MOESM1_ESM.docx]

**Table S1** Clinical characteristics of patients stratified by glypican-3 expression

| **Characteristic** | **Glypican-3 positive (n=251)** | **Glypican-3 negative (n=65)** | ***P* Value** |
| --- | --- | --- | --- |
| Age (years) | 59 (51-67) | 60 (55-68) | 0.18 |
| Sex |  |  | 0.24 |
| Male | 209 (83.27%) | 58 (89.23%) |  |
| Female | 42 (16.73%) | 7 (10.77%) |  |
| Hepatitis B |  |  | 0.96 |
| Present | 200 (79.68%) | 52 (80.00%) |  |
| Absent | 51 (20.32%) | 13 (20.00%) |  |
| Hepatitis C |  |  | 0.18 |
| Present | 25 (9.96%) | 3 (4.62%) |  |
| Absent | 226 (90.04%) | 62 (95.38%) |  |
| Steatohepatitis |  |  | 0.52 |
| Present | 11 (4.38%) | 4 (6.15%) |  |
| Absent | 240 (95.62%) | 61 (93.85%) |  |
| Alcoholism |  |  | 0.50 |
| Present | 10 (3.98%) | 4 (6.15%) |  |
| Absent | 241(96.02%) | 61 (93.85%) |  |
| Cirrhosis |  |  | 0.91 |
| Present | 168 (66.93%) | 43 (66.15%) |  |
| Absent | 83 (33.07%) | 22 (33.85%) |  |
| Status |  |  | 0.77 |
| Primary | 237 (94.42%) | 61 (93.85%) |  |
| Recurrent | 14 (5.58%) | 4 (6.15%) |  |
| Child-Pugh |  |  | 0.13 |
| A | 245 (97.61%) | 61 (93.85%) |  |
| B | 6 (2.39%) | 4 (6.15%) |  |
| Barcelona Clinic Liver Cancer |  |  | 0.17 |
| 0 | 43 (17.13%) | 6 (9.23%) |  |
| A | 53 (21.12%) | 19 (29.23%) |  |
| B | 155 (61.75%) | 40 (61.54%) |  |
| Alpha-fetoprotein (μg/L) | 19.43 (3.54-279.30) | 3.84 (2.70-18.28) | < 0.001 |

Note: Quantitative data are median (interquartile range).
